# Taguchi S/N and TOPSIS Based Optimization of Fused Deposition Modelling and Vapor Finishing Process for Manufacturing of ABS Plastic Parts

**DOI:** 10.3390/ma13225176

**Published:** 2020-11-17

**Authors:** Jasgurpreet Singh Chohan, Raman Kumar, TH Bhatia Singh, Sandeep Singh, Shubham Sharma, Jujhar Singh, Mozammel Mia, Danil Yurievich Pimenov, Somnath Chattopadhyaya, Shashi Prakash Dwivedi, Wojciech Kapłonek

**Affiliations:** 1 Department of Mechanical Engineering, UCRD, Chandigarh University, Mohali 140413, India; jaskhera@gmail.com (J.S.C.); ramankakkar@gmail.com (R.K.); thokchombhatiasingh@gmail.com (T.H.B.S.); drsandeep1786@gmail.com (S.S.); 2 Department of Mechanical Engineering, IK Gujral Punjab Technical University, Kapurthala 144603, India or shubhamsharmacsirclri@gmail.com (S.S.); jujharsing2085@gmail.com (J.S.); 3 Department of Mechanical Engineering, Imperial College London, Exhibition Rd., London SW7 2AZ, UK; 4 Department of Automated Mechanical Engineering, South Ural State University, Lenin Prosp. 76, Chelyabinsk 454080, Russia; danil_u@rambler.ru; 5 Department of Mechanical Engineering, Indian Institute of Technology (Indian School of Mines), Dhanbad, Jharkhand 826004, India; somnathchattopadhyaya@iitism.ac.in; 6 Department of Mechanical Engineering, G.L. Bajaj Institute of Technology and Management, Greater Noida 201308, India; spdglb@gmail.com; 7 Department of Production Engineering, Faculty of Mechanical Engineering, Koszalin University of Technology, Racławicka 15-17, 75-620 Koszalin, Poland; wojciech.kaplonek@tu.koszalin.pl

**Keywords:** FDM, tensile strength, vapour finishing, ABS, weight gain

## Abstract

Despite several additive manufacturing techniques are commercially available in market, Fused Deposition Modeling (FDM) is increasingly used by researchers and engineers for new product development. FDM is an established process with a plethora of advantages, but the visible surface roughness (SR), being an intrinsic limitation, is major barrier against utilization of fabricated parts for practical applications. In the present study, the chemical finishing method, using vapour of acetone mixed with heated air, is being used. The combined impact of orientation angle, finishing temperature and finishing time has been studied using Taguchi and ANOVA, whereas multi-criteria optimization is performed using the Technique for Order of Preference by Similarity to Ideal Solution (TOPSIS). The surface finish was highly responsive to increase in temperature while orientation angle of 0° yielded maximum strength; increase in finishing time led to weight gain of FDM parts. As the temperature increases, the percentage change in surface roughness increases as higher temperature assists the melt down process. On the other hand, anisotropic behaviour plays a major role during tensile testing. The Signal-to-noise (S/N) ratio plots, and ANOVA results indicated that surface finish is directly proportionate to finishing time because a longer exposure results in complete layer reflowing and settlement.

## 1. Introduction

**Figure 1:**
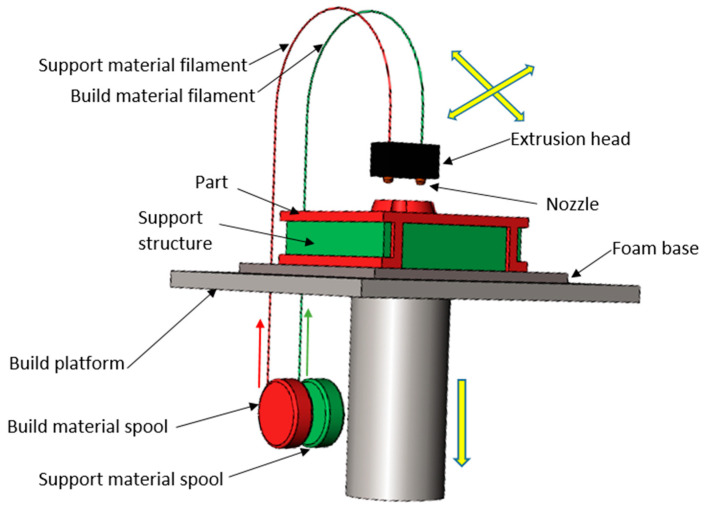
Schematic of Fused Deposition Modeling.

Additive manufacturing is a digital fabrication process used for the fabrication of tailor-made parts utilizing principle of layer-by-layer material addition [1]. The process finds vast applications in automobile, aerospace, military, and medical sector for direct use and used for secondary applications such as moulding, casting, die making [2,3]. Research and development in these industries depends extremely on this technology; staying in a place to create test designs very easily is key and old methods may have a sizable degree of time. Fused Deposition Modeling (FDM) is one of most popular additive manufacturing techniques due to its simplicity, flexibility, portability, and low production cost. In FDM, digital drawing (CAD file) of product is converted into stereolithography (STL) format which is further transferred to machine. These data files are segmented into vast layers by computer software, these are then processed to generate the tool paths [4]. The FDM comprises of heated nozzle which is capable to move in X, Y and Z directions through numerically controlled servo motors (Figure 1). 

The thermoplastic part material and support material in the form of thin wire is fed by rollers into heated nozzle which partially melts the wire. As the nozzle moves in a pre-decided path in X, Y and D directions, the thermoplastic layers are deposited on table. This layer by layer deposition of plastic material created an actual product in extremely small duration (as compared to conventional manufacturing techniques) depending upon size and shape. The overhanging parts are made of support material which can be simply cleaned after manufacturing [5]. Despite vast applications and advantages, FDM technology has certain process limitations due to its inherent behaviour of layer deposition. This leads to roughness and stair-stepping on upper surface which on one hand distorts the aesthetic value of parts, but also hinder its applicability where accuracy and finish are desirable.

Chohan et al. [6] explored various strategies adopted to improve the surface-quality of printed parts and found that various optimization studies yielded different results based on part geometry and intricacy. Therefore, it was deduced that adaptive slicing and parameter optimization are not a reliable method to achieve best finish for every case. Therefore, researchers concentrated their experiments on the use of post-processing techniques as the finish cannot be improved beyond one limit using pre-processing techniques. Sood et al. [7] studied the impact of five FDM process parameters and found that with an addition of number of layers and reduction in layer thickness, the strength improves due to higher bonding strength. As layer thickness is reduced, more layers are accommodated within constant volume as compared to larger layer thickness. Moreover, with an increase in layer thickness, due to stress accumulation, strength is reduced. On the other hand, small raster angle and thick raster formation improves bond strength which increases strength. Tiwary et al. [8] extended the study for medical applications by developing in-house experimental set-up for vapour treatment of hip implant patterns which would be further used for rapid casting of metal implants. A higher role of the chemical type and its concentration was observed, and its was found that 1,2 dichloroethane with maximum concentration yielded minimum roughness. Boschetto et al. [9] implemented barrel finishing of FDM parts which reduced the surface roughness significantly, but dimensional stability was impacted along with edge-cutting due to harsh effect of abrasives. Similarly, it was found that ball burnishing and vibratory-grinding processes are effective mass-finishing processes, but the dimensional accuracy was compromised, and thus, the practical usability of parts was comparatively reduced. Kumar et al. [10] utilized ball end magnetorheological process for nano finishing of FDM parts and significant impact of iron particle and abrasives concentration was found on surface roughness of finished parts. Optimization study recommended that 16.17% volume of abrasive particles, 25% volume of EIP, and 58.83% volume of distilled-water as the carrier-fluid must be used to achieve maximum surface finish (up to 81 nm).

Takagishi and Umezu [11] studied chemical melting finishing on three-dimensional (3D) printed parts with a free-form profile and concluded that the procedure is an effective alternative to traditional cleaning and painting treatment. Valerga et al. [12] reported decrease in tensile strength and hardness of PLA parts after chemical exposure. Also, the shore D hardness of test samples decreases with crystallinity. As compared to tetrahydrofuran, dichloromethane and chloroform, the ethyl acetate showed favourable results in terms of tensile strength and hardness. Neff et al. [13] studied the influence of vapour polishing on the mechanical characterisitcs and surface-roughness of the FDM printed components. A comparison of various mechanical properties such as surface roughness, mechanical strength etc. of vapour finished FDM printed parts of varying thickness was made. It was found that vapour finishing has a greater influence on thinner components and strength reduces significantly. Galantucci et al. [14] focused on tensile strength and bending strength during chemical finishing process. Part was dipped into a chemical (90% acetone and 10% water) solution for different durations and then kept in vacuum atmosphere for 1 h. In addition to surface enhancement, the bending strength of exposed parts was improved as compared to untreated samples. Also, minute reduction in tensile strength was noticed with an increase in exposure time due to erosion of upper layers by chemical. Colpani et al. [15] performed post-treatment of FDM parts with cold-vapours of dimethyl-ketone for enhancing the surface finish and 98% improvement was experienced, in terms of surface finish. Xu et al. [16] designed and tested a low-cost portable equipment for chemical treatment of printed parts. Although, the design is efficient for enhancing the surface-quality of components, temperature and flow control of vapours is not possible. Although, optimization of process parameters of FDM has significant impact on surface roughness, but the practical problems are faced when complex geometries are under investigation. However, Mechanical methods of surface finishing of FDM parts are partially successful to reduce surface roughness in many cases. The main disadvantage of mechanical method is edge-cutting and accidental breakage of complex specifics of components which prevents its serviceability for practical purposes. Therefore, a non-traditional finishing technique has been proposed where solvent vapours are used for surface enhancement of printed parts. The solvents are volatile chemicals, such as acetone, which tend to dissolve upper layers of Acrylonitrile-Butadlene-Styrene (ABS) parts when brought into contact. The direct immersion of FDM parts in acetone liquid leads to erosion of fine edges and profile of parts whereas exposure of hot vapours resulted in absorption of chemical into the part due to high porosity. This increases weight of part and reduces dimensional stability as absorbed chemical fumes leads to expansion of polymers.

**Figure 2:**
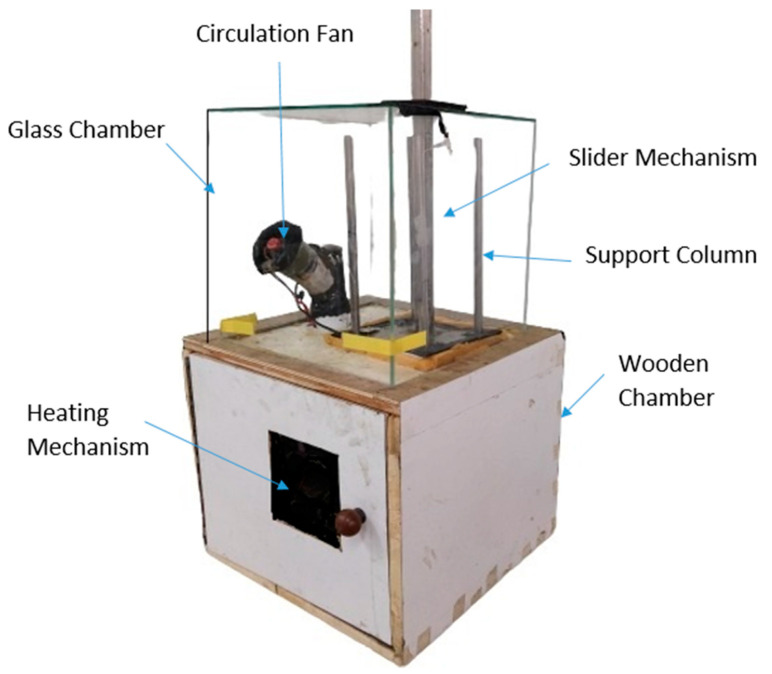
Schematic of vapour finishing apparatus.

To overcome such problems, an innovative experimental set-up has been developed for surface improvement of FDM components through hot air mixed with acetone vapours (Figure 2). 

Although experiments have been performed using in-house developed apparatus [16], the impact of temperature has never been studied. The apparatus heats air and acetone up to desired temperature and mixes in equal proportion before forcing the mixture into closed chamber where FDM parts are hanged. The present study aims to analyse the impact of various process parameters on surface-finish, tensile strength, and weight gain of FDM parts using Taguchi and ANOVA analysis for rapid casting and end-use applications. Moreover, the optimum set of process parameters for FDM and vapour finishing can be used further for mass finishing of customized products. The prime novelty in this research work is in carrying out all the experimentation using a newly-designed, dedicated vapor-finishing set-up, which is further made with provision of temperature-control to investigate the impact of operating parameters on the response-characteristics of ABS parts. Moreover, the study would use TOPSIS technique to identify the optimum parametric combination to achieve maximum surface finish, tensile strength and minimum weight gain of FDM parts.

## 2. Materials and Methods 

### 2.1. Vapor Finishing and FDM Apparatus

A vapour finishing test-rig have designed and fabricated which has provision to alter temperature of chemical fumes. Moreover, the innovative apparatus has been designed to avoid wastage of chemical vapours during loading and unloading of FDM Parts, as shown in Figure 2. For testing the efficacy of apparatus and parametric optimization, ABS material (Grade: P400), supplied by Stratasys Ltd., Eden Prairie, Minnesota, Inc., USA is used for fabrication of samples using Cube Pro commercial FDM printer (Make: 3D Systems, Rock Hill, South Carolina (SC), USA). The specimen is first designed as a CAD model in a computer according to ASTM standard D638–14 [17] and then converted into STL format for further processing. Taguchi L9 orthogonal array (Table 1) has been designed for investigating the impact of three input factors, i.e., orientation angle, finishing time and finishing temperature which are further varied at three levels. The tensile behaviour of 3D printed parts is dependent on numerous process parameters, such a density, orientation, infill pattern and anisotropic behaviour of parts. Moreover, the strength of ABS parts manufactured by different processing techniques is different from standard values (13 MPa–65 MPa) as mentioned in product catalogue [18]. In present study, the samples are printed at 60% density, honeycomb internal structure, 10 mm/s extrusion speed and 0.1 mm layer thickness. 

For present experimentation total twenty-seven parts are fabricated to carry out nine experiments taking three repetitions for respectively trials to evade random error. The specimens are fabricated at 0°, 45° and 90° orientation angle as previous literature revels that these settings have maximum impact on surface finish and tensile strength. After fabrication, the printed specimens are taken out of print bed carefully and treated in ultrasonic bath for cleaning and removal of support structure.

### 2.2. Measurement Equipment and Design of Experiments

The average surface roughness of FDM parts was calculated through Profile Measurement Equipment “SJ-210” (Make: Mitutoyo Corporation, Kawasaki-shi, Japan). The average-roughness (Ra) is specifically examined for current investigation and observations were taken normal to deposition direction. The measurements were recorded using stylus tip angle of 60 °C tip and radius 2μm moving at 0.75 mN. The Gaussian filter was used for filtering the surface roughness profiles at cut off length 0.025 cm and 0.25 cm transverse length as per ISO 4287 [19]. The surface roughness of each sample was measured three times and average of three readings was considered as final value to avoid error. 

The mass of samples has been calculated before and after finishing using Citizen digital weighing apparatus (Make: Citizen Industries Co. Ltd., Mumbai, India) having readability upto 0.0001 g and maximum limit of 220 g. During weight and roughness measurements, three readings were recorded, each pre- and post-finishing, and the average of three readings was taken as the final value. Moreover, surface-roughness was deliberately evaluated at the same location before, and after, the finishing process to avoid random errors. 

For Taguchi analysis, percentage-variation in weight and surface-roughness are used as response parameters and calculated as following Equation (1):

(1) $$\mathrm{Percent}\;\mathrm{change}=\left[\frac{\left(\mathrm{Initial}\;\mathrm{measurement}\;-\;\mathrm{Final}\;\mathrm{measurement}\right)}{\mathrm{Initial}}\mathrm{measurement}\right]\times 100.$$

**Figure 3:**
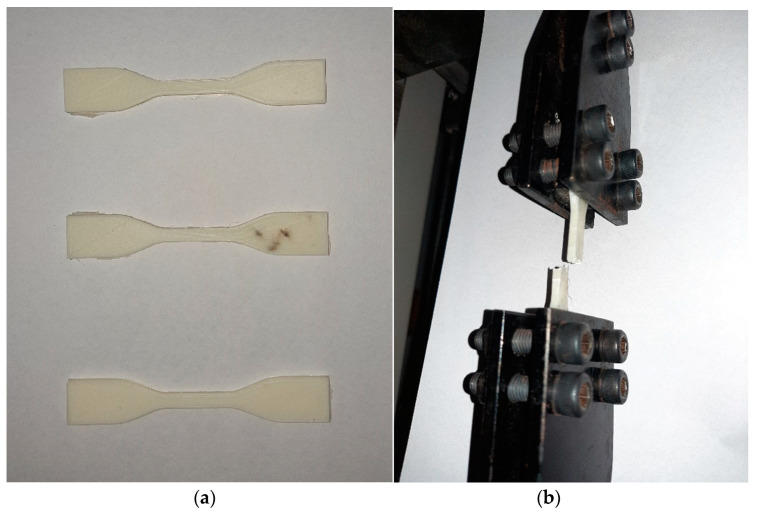
(**a**) Tensile testing of finished samples (**b**) Sample prepared through FDM process.

After finishing process, the peak tensile strength of parts was measured using Universal Tensile Tester (supplied by Shanta Engineering, Pune, India) with 500 mm/min cross head movement speed as per ASTM 638-14 standards [17] as shown in Figure 3. Nine experiments were performed with three replications of each experiment which lead to total twenty-seven samples under observation. 

For percentage-variation in surface-roughness and tensile strength, the “larger is better” characteristic is required and thus, Signal to Noise Ratio (SN Ratio) is computed [20] using Equation (2):

(2) $$\mathrm{SN}\;\mathrm{Ratio}\;=\;-10\;\mathrm{Log}\;\left[\frac{1}{n}\;\sum_{i=1}^{n}1/Y_{i}^{2}\right].$$

Whereas, the “smaller is better” characteristic is required for percentage change in weight and SN Ratio is calculated in Equation (3):

(3) $$\;\mathrm{SN}\;\mathrm{Ratio}\;=\;-10\;\mathrm{Log}\;\left[\frac{1}{n}\;\sum_{i=1}^{n}Y_{i}^{2}\right].$$

In above equation, n represents number of replicated experiments while i is response which may vary from 1 to n.

The micrographs were obtained to ascertain the manifestations occurring at micro scale on the surface and in cross-section of FDM parts. The micrographs are retrieved using Scanning Electron Microscope (SEM) apparatus (Model: IT500, Make: Jeol Ltd., Tokyo, Japan). Tensile strength determines the maximum stress that a material can withstand without breaking. Hence, the images of failure point are taken at high vacuum mode, 10 KV accelerating voltage, 12 mm. working distance and double sided carbon tape. Initially, the site of the break point, five millimeters of the cross section was cut and subjected to gold coating to make it conductive. The coated parts were then placed in a vacuum chamber where the scanning of the parts at different magnification takes place.

## 3. Results and Discussions

### 3.1. Taguchi and ANOVA Analysis

The Taguchi L9 orthogonal-array along with Signal to Noise (SN) Ratio of leading and trailing observations of surface-roughness, tensile strength and weight has been calculated. It can be clearly noticed that the percentage-variation in average surface roughness (%∆Ra) varies from 64.12% to 87.59% after vapor finishing. Table 2 displays ANOVA test results for surface roughness.

The maximum contribution (84.88%) of finishing temperature has been observed at 95% confidence level. The higher temperature ensures effective meltdown of upper layers of ABS polymer which intensified the surface enhancement phenomenon after parts are cooled. On the other hand, orientation angle does not affect the surface roughness as vapours could not to reach inside layers of printed polymer parts. Similarly, finishing time does not influence the surface finishing phenomenon as hot vapours instantly melted the upper layers after exposure. The measurements of surface-roughness also indicated that the percentage change in surface-roughness through vapor finishing is significantly more than other mechanical finishing techniques as well as pre-processing techniques [6]. 

The tensile strength results were retrieved and percentage contribution of each parameter has been displayed in Table 3 along with individual *P*-values and *F*-values. It can be deduced from *F*-values and *P*-values of ANOVA table that orientation angle significantly affects the tensile strength at 95% confidence level. The internal polymer bead deposition angle enhances the strength of parts in certain direction due to phenomenon of anisotropy in FDM parts.

In case of weight measurements, the average weight of FDM samples has increased even after cooling down the parts for 24 h after chemical vapor exposure. In present experimentation, the percentage gain in weight ranges from 0.14% to 0.40%, for all the experiments. Table 4 shows the ANOVA test results for percentage change in weight.

**Figure 4:**
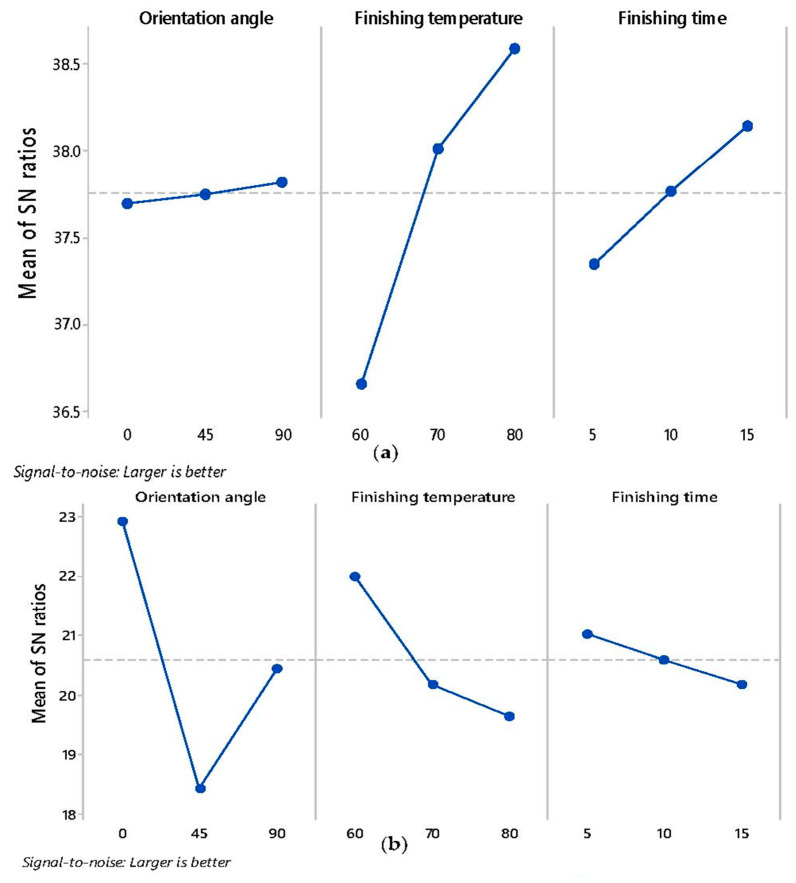

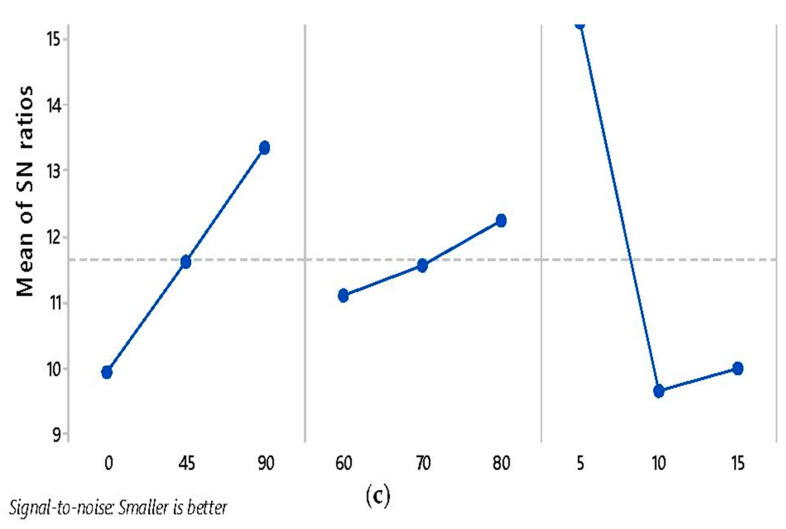
Main-effects plot for S/N-ratio of (**a**) Percent-variation in Surface-roughness (**b**) tensile Strength (**c**) Percentage change in weight.

Figure 4 plots the main-effects graph for S/N-ratios of each response-parameter i.e., surface-roughness, tensile strength, and weight of FDM parts. As evident in the trend lines, the most substantial parameter which affects the surface roughness is finishing temperature, followed by finishing time. The orientation angle of FDM parts does not impact the surface roughness as vapor finishing process completely eliminates the effect of layer deposition at different angles. The increase in finishing temperature and time both resulted in higher SN ratio, which indicates a higher surface finish. Colpani et al. [21] also reported that minimum surface roughness was achieved for ABS molds fabricated at 90° orientation angle during 120-min exposure and 12-h drying time. Another study found orientation angle of 60° favourable for surface finish during dichloromethane exposure [22]. Conversely, lower levels of finishing time and temperature resulted in higher tensile strength. However, these parameters have lesser impact on tensile strength of FDM parts as compared to orientation angle as suggested by ANOVA tables and SN ratio plots. The layer deposition strategy at different angles strengthens the inter-layer bonding in specific direction leading to anisotropic behavior of FDM part [6]. Therefore, in the present case, parts manufactured at orientation of 0° and finished at lower temperature and time yielded maximum peak strength because FDM printer deposited the rasters in longitudinal direction.

On other hand, 45° orientation have least strength due to weak bonding between layers fabricated at this angle. Although peak strength achieved at 90° orientation angle is greater than 45°, but due to change in deposition strategy, it could not yield maximum strength. Furthermore, the weight of FDM samples is directly proportional to finishing time, which is quite evident because with an increase in finishing time results in a higher exposure, which resulted in more absorption of vapors inside the FDM parts which resulted in weight gain. On the other hand, finishing temperature and orientation angle have least impact on the weight gain.

### 3.2. SEM Micrograph and Surface Profile Analysis

**Figure 5:**
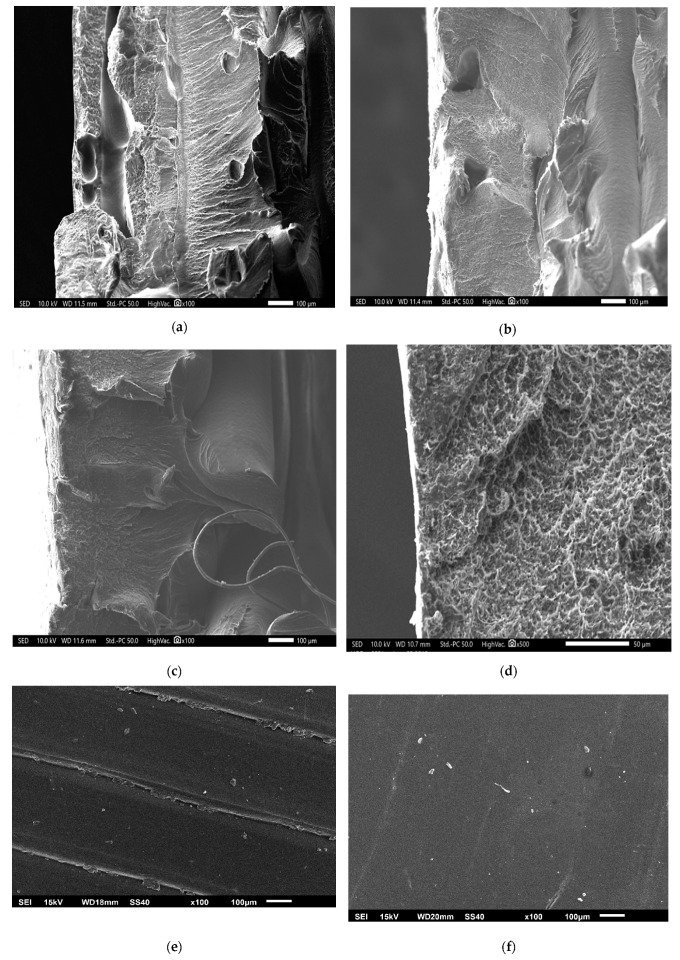
SEM Micrographs of Specimen, (**a**) Point of failure of tensile-fractured sample 1; (**b**) Point of failure of tensile-fractured sample 9; (**c**) Point of failure of tensile-fractured sample 6; (**d**) Internal surface of sample 1; (**e**) upper view before finishing of sample 1 and (**f**) upper view after finishing of sample 1.

Figure 5a–c shows the SEM images at point of fracture of FDM parts subjected to tensile tests. The micrographs of sample 1 (manufactured at 0° orientation angle and 60° temperature) and sample 6 (manufactured at 45° orientation angle and 80° temperature) have been acquired, which showed a maximum, and minimum peak tensile strength, respectively to evaluate the breakage behaviour of FDM parts. As seen in images, internal surface of sample 1 shows higher surface integrity and uniformity as compared to 45° orientation angle. Moreover, the impact of finishing temperature can be clearly seen which concludes that vapours even enter into internal sections of FDM parts. During exposure, the vapours at lower temperature manifests lesser changes in internal bonding and thus tensile strength is retained. On the other hand, samples made at 45° orientation angle does not retain its strength due to high temperature exposure and anisotropic behaviour of FDM parts.

**Figure 6:**
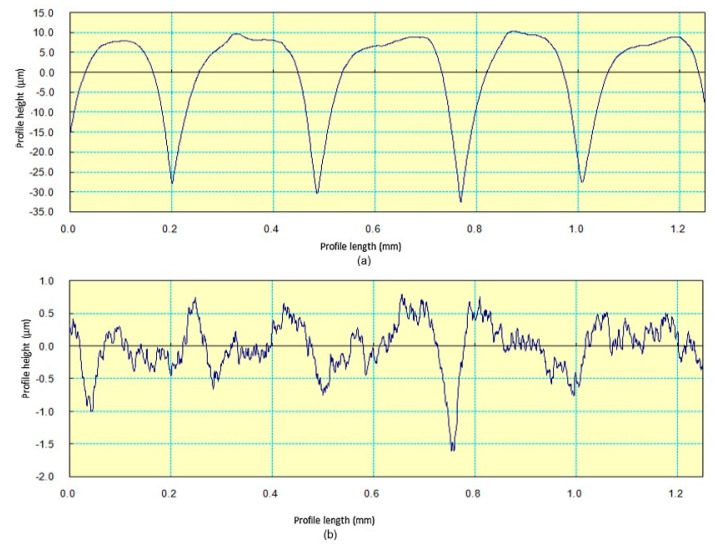
Surface Roughness Profiles of Specimen (**a**) before finishing (**b**) after finishing.

Figure 5d–f compares the upper surface of FDM samples before finishing and after finishing respectively. The peaks and valleys formed due to layer-by-layer deposition are clearly seen as rough surface before finishing. When hot vapors of acetone come in contact with the ABS material, the upper layers melt partially and starts flowing like thick fluid during exposure. Afterwards, the parts are cooled down where materials from peaks of layer flows down into valley and settle down as smooth surface due to surface energy. This phenomenon of finishing is supported by Taguchi results where finishing temperature is significant parameter for surface roughness. As temperature increases, the percentage change in surface roughness increases because higher temperatures assist the melt down process. Similarly, the SN ratio plot indicated that surface finish is directly proportional to finishing time because longer exposure results in complete layer reflowing and settlement [21]. A similar phenomenon has been deduced from surface roughness profiles of FDM samples under study. The surface-roughness profile of FDM parts pre- and post-finishing are shown in Figure 6a,b respectively. The semi-circular bead of ABS filament deposited by FDM extruder head has been plotted in Figure 6a by surface roughness tester. Afterwards, the materials reflow and settle down as smooth surface as peak height is significantly reduced in Figure 6b. Moreover, after finishing the circular shape of material is lost due to material melt down and changes in zig zag shapes as materials fills the voids on upper surface of FDM parts. Similar layer smoothing phenomenon has been found by Panda et al. [23] during chemical dipping and vapour exposure of PLA part using dichloromethane. Although, the average surface-roughness is significantly reduced after chemical vapor finishing as experienced by other researchers using dedicated finishing apparatus [16,24].

### 3.3. Multi-Criteria Optimization

**Figure 7:**
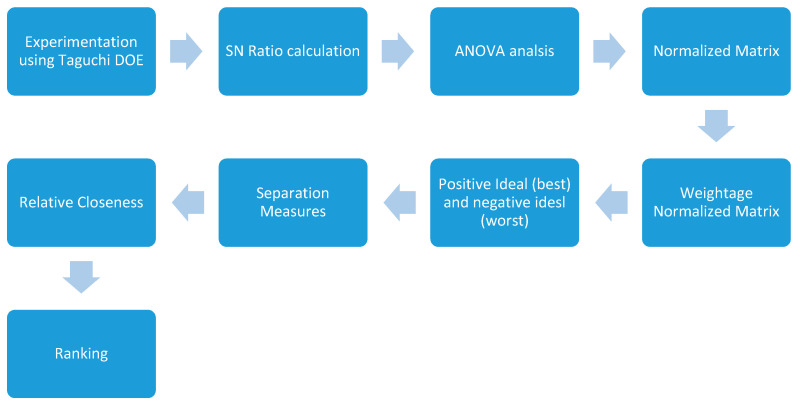
Process flow chart of implementation of TOPSIS for Multi-attribute optimization of process parameters.

Although, an optimization study has been conducted to analyze the impact of individual parameter on response, there is requirement for a multi-criteria optimization, which would yield the best results for every response. The present work objects to evaluate the optimum settings of FDM and finishing processes which would equally balance all the responses. Therefore, TOPSIS tool has been used to identify the optimized parameter settings was sub sequentially proposed [25]. As suggested by this method, the best alternative will be closest to the positive-ideal solution and at maximum distance from the negative ideal solution [26]. The detailed process flow chart of implementation of TOPSIS has been shown in Figure 7. The TOPSIS tool utilizes existing SN ratios calculated after experimentation for multi-criteria optimization using below mentioned five steps.

#### 3.3.1. Normalized Matrix

Initially, the values of normalized matrix for experimentation abbreviated as rij are calculated using Equation (4) given below:

(4) $$r_{\mathrm{ij}}\;=\;\frac{a_{\mathrm{ij}}}{{\left(\sum_{i=1}^{n}a_{\mathrm{ij}}\right)}^{0.5}}.$$

#### 3.3.2. Weightage Normalized Matrix

In second step, the weights of response parameters are multiplied with respective columns of normalized matrix. In present work, the equal importance has been given to each response parameter. The values of normalized matrix weightage normalized matrix have been shown in Table 5.

#### 3.3.3. Evaluation of the Positive-Ideal (Best) and Negative-Ideal (Worst) Solutions

The positive-ideal (generally the best solution) and negative-ideal (generally the worst solution) can be written as Equations (5), and (6), respectively, ℇ

(5) $$V^{+}=\;\left\{\langle \;max\left(t_{ij\;}\;|\;i=1,2,\;\ldots .m\right)\;|\;j\;ℇ\;J_{-}\;\rangle ,\;\langle min(t_{ij\;}|\;i=1,2,\;\ldots \ldots ,m)\;|\;j\;ℇ\;J_{+}\rangle \right\}$$

(6) $$V^{-}=\;\left\{\langle \;min\left(t_{ij\;}\;|\;i=1,2,\;\ldots .m\right)\;|\;j\;ℇ\;J_{-}\;\rangle ,\;\langle max(t_{ij\;}|\;i=1,2,\;\ldots \ldots ,m)\;|\;j\;ℇ\;J_{+}\rangle \right\}$$

$J_{+}\;=\;\left\{j=1,2,\ldots \ldots m\;|\;j\;\right\}$ associated with the criteria for beneficial attribute

$J_{-}\;=\;\left\{j=1,2,\ldots \ldots m\;|\;j\;\right\}$ associated with the criteria for non-beneficial attribute

The evaluated values of positive-ideal (best) and negative-ideal (worst) results are displayed in Table 6.

#### 3.3.4. Evaluation of the Separation Measures

Euclidean distance concept is utilized to evaluate the value of positive and negative separation measures. Hence, Equations (7) and (8) are used to compute the separation measures,

(7) $$S_{1}^{+}=\;{\left\{\overset{m}{\underset{j=1}{\sum }}{\left(V_{ij}-V_{j}^{+}\right)}^{2}\right\}}^{0.5}$$

(8) $$S_{1}^{-}=\;{\left\{\overset{m}{\underset{j=1}{\sum }}{\left(V_{ij}-V_{j}^{-}\right)}^{2}\right\}}^{0.5}$$

The solutions calculated after implementing afore-mentioned equations have been shown in Table 7.

#### 3.3.5. Evaluation of the Relative Closeness

The ranking of alternates is done based on the relative closeness-value. The relative closeness of alternative Aj is written in Equation (9):

(9) $$C_{j}\;=\;\frac{S_{j^{-}}}{\left(S_{j^{-}\;}+\;S_{j^{+}}\right)}$$

Finally, Equation (9) has been used for calculation of relative closeness along with ranking for each experimental result has been shown in Table 8. It can be deduced that experiment no. 1 has maximum relative closeness followed by experiment no. 7 and 8. Therefore, the parameter settings of experiment 1 have been recommended for finishing of FDM parts to attain maximum output from each response, i.e., surface roughness, tensile strength and weight of FDM parts. It is, thus, recommended to execute the FDM process at 0° orientation with 60° finishing temperature and 5-min exposure duration. 

The optimization study has been exclusively conducted to ascertain the effect of combined operating parameters of FDM and vapour finishing process utilizing dedicated apparatus which creates controlled environment for vapour finishing. An apparatus has been prepared which utilizes air at variable temperature mixed with chemical vapours. TOPSIS have utilized for multi-objective optimization of process parameters which is highly effective tool used for industrial applications.

## 4. Conclusions

The study aims to investigate the impact of the orientation angle, finishing temperature and time on surface quality, tensile strength and weight gain of FDM parts during surface finishing process. A dedicated experimental set-up has been designed and made with provision of temperature control to investigate the impact of operating parameters on surface finish, tensile strength and weight of ABS parts. TOPSIS has been applied for multi-criteria optimization of FDM components to acquire single set of process parameters for mass production of finished FDM parts. Moreover, the optimal-set of process parameters for FDM and vapour finishing can be used for mass finishing of customized products. The study can be further broadened to evaluate the impact of process-parameters on dimensional accuracy, compressive strength and wear behaviour. Following conclusions are derived:

- The study has been conducted on dedicated vapor finishing apparatus which utilized influence of hot chemical vapors mixed with heated air for surface enhancement of ABS parts.
- The finishing temperature has a significant impact on surface finish, while orientation angle and finishing time were significant parameters for tensile strength and weight of FDM parts respectively.
- It was found that higher temperature resulted in better finish due to instant meltdown of upper plastic layers.
- Orientation angle of 0° led to highest value of tensile strength as layers are deposited in horizontal plane. Moreover, higher exposure duration induced permanent weight gain due to increase in absorption of vapours.
- Since each response parameter is impacted differently by input parameters, multi-criteria optimization tool TOPSIS has been utilized to identify optimal settings.
- The optimum parameter settings can be implemented to improve the surface-quality of FDM parts which can be utilized for end-use products and for rapid tooling applications.

## Figures and Tables

**Table 1:** Experimental control log with SN ratio of each response.

Exp. No.	Input Parameters			Surface Roughness		Tensile Strength		Weight	
	A (°)	B (°C)	C (min)	%∆R_a_	SN Ratio	Peak Strength (MPa)	SN Ratio	%∆w	SN Ratio
**1**	0	60	5	64.12	36.1399	16.75	24.4803	0.23	−14.53
**2**	0	70	10	80.35	38.0997	14.09	22.9782	0.35	−18.47
**3**	0	80	15	87.59	38.8491	11.67	21.3414	0.40	−19.44
**4**	45	60	10	67.54	36.5912	9.57	19.6182	0.39	−18.81
**5**	45	70	15	82.64	38.3438	7.33	17.3021	0.33	−17.53
**6**	45	80	5	82.25	38.3027	8.3	18.3816	0.14	−9.79
**7**	90	60	15	72.78	37.2402	12.45	21.9034	0.24	−12.78
**8**	90	70	5	75.83	37.5968	10.28	20.2399	0.16	−9.77
**9**	90	80	10	85.31	38.6200	9.12	19.1999	0.26	−13.99

A = Orientation Angle; B = Finishing Temperature; C = Finishing Time.

**Table 2:** Results of ANOVA tests for surface roughness.

Parameters	Dof	Seq. SS	Seq. MS	*F*-Value	*P*-Value	Contribution	Significance
**A**	2	0.02289	0.01145	0.31	0.763	0.32%	No
**B**	2	5.91115	2.95557	80.41	0.012	84.88%	Yes
**C**	2	0.95623	0.47811	13.01	0.071	13.73%	Yes
**Error**	2	0.07351	0.03676			0.10%	
**Total**	8	6.96378					

A = Orientation Angle; B = Finishing Temperature; C = Finishing Time; Dof = Degrees of freedom; SS = Sum of squares; MS = Mean squares.

**Table 3:** Results of ANOVA tests for tensile strength.

Parameters	Dof	Seq. SS	Seq. MS	*F*-Value	*P*-Value	Contribution	Significance
**A**	2	30.478	15.2388	28.85	0.034	72.89%	Yes
**B**	2	9.190	4.5952	8.70	0.103	21.97%	No
**C**	2	1.088	0.5440	1.03	0.493	2.60%	No
**Error**	2	1.056	0.5282			2.52%	
**Total**	8	41.813					

A = Orientation Angle; B = Finishing Temperature; C = Finishing Time; Dof = Degrees of freedom; SS = Sum of squares; MS = Mean squares.

**Table 4:** Results of ANOVA tests for weight change.

Parameter	Dof	Seq. SS	Seq. MS	*F*-Value	*P*-Value	Contribution	Significance
**A**	2	7.0879	3.5440	53.98	0.018	7.49	No
**B**	2	6.6804	3.3402	50.88	0.019	7.06	No
**C**	2	80.6525	40.3264	614.23	0.002	85.29	Yes
**Error**	2	0.1313	0.0657			0.13	
**Total**	8	94.5524					

A = Orientation Angle; B = Finishing Temperature; C = Finishing Time; Dof = Degrees of freedom; SS = Sum of squares; MS = Mean squares.

**Table 5:** Values of normalized and weightage normalized matrix of response parameters.

	Normalized Matrix of Response Parameters			Weightage Normalized Matrix of Response Parameters		
S. No.	Weight	Tensile Strength	Surface Roughness	Weight	Tensile Strength	Surface Roughness
1	0.263	0.489	0.274	0.088	0.163	0.091
2	0.400	0.411	0.343	0.133	0.137	0.114
3	0.457	0.341	0.374	0.152	0.114	0.125
4	0.445	0.279	0.289	0.148	0.093	0.096
5	0.377	0.214	0.353	0.126	0.071	0.118
6	0.1	0.242	0.352	0.053	0.081	0.117
7	0.274	0.363	0.311	0.091	0.121	0.104
8	0.183	0.300	0.324	0.061	0.100	0.108
9	0.297	0.266	0.365	0.099	0.089	0.122

**Table 6:** Values of positive ideal and negative ideal solutions.

Output Parameters	Positive-Ideal	Negative-Ideal
Weight	0.053	0.152
Tensile strength	0.163	0.071
Surface roughness	0.125	0.091

**Table 7:** Values of positive and negative separation measure.

Experiment No.	Positive Separation Measure	Negative Separation Measure
**1**	0.048	0.112
**2**	0.085	0.072
**3**	0.111	0.054
**4**	0.121	0.023
**5**	0.117	0.038
**6**	0.083	0.103
**7**	0.060	0.080
**8**	0.066	0.097
**9**	0.087	0.064

**Table 8:** Values of relative closeness and ranking.

Exp. No.	C_ij_	Individual Rank
1	0.701	1
2	0.461	5
3	0.327	7
4	0.157	9
5	0.243	8
6	0.554	4
7	0.569	3
8	0.597	2
9	0.422	6
